# HLA-G Orchestrates the Early Interaction of Human Trophoblasts with the Maternal Niche

**DOI:** 10.3389/fimmu.2015.00128

**Published:** 2015-03-30

**Authors:** Silvia Gregori, Giada Amodio, Federica Quattrone, Paola Panina-Bordignon

**Affiliations:** ^1^Division of Regenerative Medicine, Stem Cells and Gene Therapy, San Raffaele Telethon Institute for Gene Therapy (HSR-TIGET), IRCCS San Raffaele Scientific Institute, Milan, Italy; ^2^Reproductive Sciences Laboratory, Division of Genetics and Cell Biology, IRCCS San Raffaele Hospital, Milan, Italy

**Keywords:** HLA-G, trophoblasts, dendritic cells, IL-10, T regulatory cells, vascular remodeling

## Abstract

Extravillous trophoblasts (EVTs) play a central role in educating maternal leukocytes, endometrial stromal and endothelial cells to generate a receptive decidual microenvironment tailored to accept the semi-allogeneic fetus. HLA-G, a non-classical HLA class I molecule endowed with immune-regulatory functions, is primarily expressed on EVTs lining the placenta and on the naturally occurring tolerogenic dendritic cells, named DC-10, which are enriched in the human first trimester decidua. Decidual DC-10 are involved in HLA-G-mediated tolerance at the maternal–fetal interface. EVTs not only establish a tolerogenic microenvironment through the interaction with maternal innate and adaptive cells but also orchestrate placenta vascular and tissue remodeling, leading to a successful pregnancy. Here, we discuss the potential implications of the HLA-G-mediated cross-talk among the cells present at the maternal–fetal interface, and its role in maintaining a positive relationship between the mother and the fetus.

## Introduction

**Figure 1:**
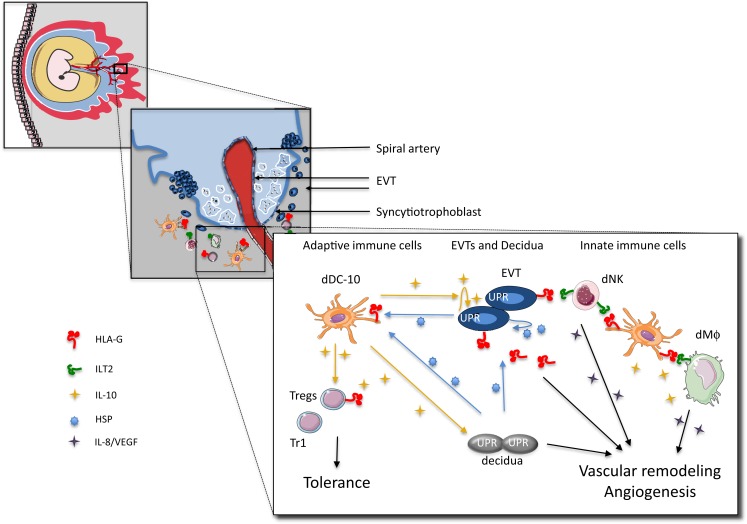
**Proposed model for cross-talk among embryo trophoblasts, decidual leukocytes, and stromal cells at the maternal–fetal interface in human first trimester pregnancy**. EVTs express and secrete HLA-G, and release IL-10 (and TSLP), which instruct dAPCs to become tolerogenic DC (i.e., dDC-10 or TSLP-modulated dDC) secreting IL-10 and promoting the induction of a variety of Tregs (i.e., Tr1 cells, CD4^+^CD25^+^FOXP3^+^ Tregs, and CD4^+^HLA-G^+^ Tregs). Induced Tregs inhibit effector T cells, and, via IL-10 secretion, promote HLA-G expression on EVTs. EVTs via HLA-G directly promote dNK cell activation and the release of angiogenic factors. dDC-10 is HLA-G^+^ and can interact with either dNKs or dMΦ via ILT2, and promote their activation and pro-angiogenic effects. dDC-10 themselves secrete also pro-angiogenic factors supporting neo-vascularization. HSPs secreted by the maternal cells and trophoblasts contribute to the regulation of HLA-G expression on dAPCs and EVTs. Finally, IL-10 modulates the UPR pathway and regulates vascular uterine remodeling by HLA-G^+^ EVTs.

The maternal–fetal interface is composed of fetal trophoblasts intermingled with maternal leukocytes, stromal, and endothelial cells that comprise the decidua. During implantation, trophoblasts, derived from the trophoectoderm surrounding the blastocyst, differentiate into the syncytiotrophoblasts that infiltrates the endometrium, and the cytotrophoblasts at the embryo side. The layer of syncytiotrophoblasts in contact with the decidua represents the extravillous trophoblasts (EVTs) (Figure 1). EVTs orchestrate bi-directional cross-talk between the mother and the fetus by providing structural and biochemical barriers, serving as an endocrine organ that support and regulate placental and fetal development and growth, and modulating maternal innate and adaptive immune responses (1).

The evidence that, after embryo implantation, defective development and function of EVTs can lead to fetal loss and pregnancy-associated pathological conditions, including pre-eclampsia and intrauterine growth restriction (2–4), sustains the important role of EVTs in orchestrating the decidual modification for successful pregnancy. The expression of HLA-G, a non-classical HLA class I molecule, on EVTs contributes to trophoblast invasiveness, decidual cell differentiation, vascular remodeling, and maintenance of a local immunosuppressive state. A proper understanding of regulatory mechanisms that control EVTs interaction with the maternal niche is a critical issue in reproduction.

## State of the Art

### Hormonal regulation at the maternal–fetal interface

The endometrial microenvironment, constituted by luminal and glandular epithelial cells, stromal cells, fibroblasts, vascular smooth muscle cells, endothelial cells, leukocytes, endometrial stem cells, and dynamic leukocyte populations, undergoes cyclical changes regulated by sex hormones. In the absence of pregnancy, the endometrium is sloughed off at menstruation. In the post-menstrual proliferative phase, under estradiol stimulation, it undergoes rapid regeneration into a fertile soil capable to accept the embryo (5). During the secretory phase, the blood flow increases, the arteries branches, and the glands enlarge and start to secrete fluids rich in glycogen used by the embryo as an energy source in its early stages of growth. These processes are driven by the post-ovulatory rise of progesterone that inhibits the pro-proliferative effect of estradiol and, in mammals, induces a radical transformation of the endometrium (pre-decidualization) that heralds the limited period of endometrial receptivity, (“implantation window”) during which embryo attachment can take place (6). Pre-decidualization is primarily defined by the transformation of endometrial stromal cells into secretory epithelioid-like decidua cells and is characterized by massive influx of maternal innate immune cells and vascular remodeling (7).

In the presence of the embryo, the human chorionic gonadotropin (hCG) sustains the full decidualization of the endometrium via stimulation of progesterone production. hCG is the most specific embryo-derived signal observed in humans and the *hCG* gene is transcribed as early as the two-cell stage (8, 9). Being released before embryo implantation, hCG also acts on endometrial cells in a paracrine way by inducing their differentiation characterized by secretion of prolactin, leukemia inhibitory factor (LIF), and IL-6 (10, 11). Furthermore, hCG promotes angiogenesis by increasing vessel sprouting of endothelial cells and secretion of vascular endothelial growth factor (VEGF) (12, 13). The immunomodulatory properties of hCG are multiple (13): it regulates decidual natural killer (dNK) cell proliferation, contributing to the remodeling of decidual spiral arterioles (14, 15); it induces CXCL8 production by monocytes (16); it influences tolerogenic dendritic cells (DCs) proliferation and differentiation (17); and it contributes to recruitment of T regulatory cells (Tregs) (18).

The pre-ovulatory peak of estrogen is important for proliferation of the uterine epithelium in preparation for implantation, while rising progesterone after ovulation is required for implantation of the embryo and decidual differentiation. Together with hCG, progesterone and estradiol are also essential for the programing of a local tolerogenic environment (19). Progesterone polarizes T-cell responses toward an anti-inflammatory phenotype, favoring T(helper)h2 while dampening Th1 and Th17 cells, and inducing Tregs via thymic stromal lymphopoietin (TSLP) (20–22). The increased concentration of progesterone at the maternal–fetal interface may play a role in regulating HLA-G gene expression (23). Progesterone induces up-regulation of HLA-G in primary cultures of first trimester cytotrophoblasts through the binding to an alternative progesterone response element in the *HLA-G* promoter (24).

Estradiol regulates the immune system by affecting T and B cells, and down regulating NK cell cytotoxicity (25). Interestingly, estradiol helps to regulate fetal tolerance during pregnancy by expanding Tregs and their suppressive function (26, 27).

Dendritic cells, by expressing specific receptors, are susceptible to stimulation with hCG, progesterone, and estradiol. Pregnancy hormones can either activate or reduce the stimulatory activity of monocyte-derived DCs. Consistent up-regulation of IL-10 production by human DCs has been observed upon stimulation with pregnancy hormones [as reviewed in Ref. (28)].

### HLA-G-expressing trophoblast at the maternal-fetal interface

HLA-G has well-recognized immunomodulatory activities, is low polymorphic [reviewed in Ref. (29)], and has limited tissue distribution [reviewed in Ref. (30)]. HLA-G was the first HLA class I molecule identified on EVTs (31). EVTs, forming the placental interface with the maternal systemic circulation, do not express HLA class I, but as they differentiate to invade the decidua and contact maternal decidual leukocytes, they begin to express HLA-G (32). All EVTs, syncytiotrophoblasts (33), interstitial and endovascular trophoblasts, and placental bed giant cells are HLA-G positive [reviewed in Ref. (34)].

By alternative splicing of the primary transcript, four membrane-bound (HLA-G1 to -G4) and three soluble (HLA-G5 to -G7) isoforms can be generated [reviewed in Ref. (35)]. In addition, a soluble isoform, named shed HLA-G1, is released after proteolytic cleavage of the membrane-bound HLA-G1 by metalloproteinases (36, 37). Through the interaction with the inhibitory receptors immunoglobulin-like transcript (ILT)2 and ILT4, and the killer immunoglobulin-like receptor (KIR)2DL4, HLA-G regulates innate and adaptive immune responses and participates in promoting tolerance [reviewed in Ref. (38)].

During the last decade, it has become evident that the expression of HLA-G on EVTs is not primarily involved in protecting the fetus from the attack by maternal cells, but it plays an important role in tissue remodeling. HLA-G expressed or secreted by EVTs controls their decidual and endovascular invasion. EVTs can express membrane-bound or shed HLA-G1, and soluble HLA-G2, -G5, and -G6 (39–43) (Table 1). Studies in placental sections demonstrated that β2m-bound HLA-G is expressed by all EVTs, whereas more distal EVTs at the invasion front express the free heavy chain (FHC) HLA-G (40). It has been proposed that the selective expression of FHC–HLA-G, which is not recognized by ILT2 (44), may limit the inhibition of dNKs while allowing these cells to secrete factors required for successful pregnancy. *In vitro* studies showed that treatment of primary trophoblasts with HLA-G5 stimulates cell invasion and increases the production of metalloproteinases and urokinase, known to remodel the endometrial extracellular matrix (45, 46). Moreover, the interaction between HLA-G on EVTs and dNKs leads to CXCL8 and CXCL10 secretion that in turn, via stimulation of CXCR1 and CXCR3, promote EVTs invasiveness (14). Thereby, HLA-G-expressing EVTs regulate decidual invasion in both autocrine and paracrine manner.

**Table 1:** **Expression pattern of HLA-G-related molecules on cells at the maternal–fetal interface**.

Cell types		HLA-G isoforms (reference)	HLA-G receptors (reference)		
			ILT2	ILT4	KIR2DL4
EVTs		HLA-G1 (39, 40)	Neg (47)	Neg (47)	n.t.
		shed HLA-G1 (40, 42)	
		HLA-G2 (42)	
		HLA-G5 (41)	
		HLA-G6 (43)	
Syncytiotrophoblasts		HLA-G5 (33)	Neg (47)	Neg (47)	n.t.
Endothelial cells	Maternal endothelium	n.t.	Neg (47)	Neg (47)	n.t.
	Fetal vessels	n.t.	Neg (47)	n.t.	n.t.
Endometrial stromal cells		n.t.	Pos (47)	Neg (47)	n.t.
dNK	Total CD56^+^	Neg (48)	Posow^low^ (49)	Neg (49)	Pos (49–51)
CD4^+^	Total CD4^+^	n.t.	Pos (52)	n.t.	Pos (52)
	CD4^+^HLA-G^+^	HLA-G1 (53, 54)	n.t.	n.t.	n.t.
CD8^+^	Total CD8^+^	n.t.	n.t.	n.t.	n.t.
	CD8^+^HLA-G^+^	HLA-G1 (53)	n.t.	n.t.	n.t.
Macrophages	CD14^+^CD163^+^	Neg (55)	Pos (50, 56)	Pos (50, 56)	n.t.
DCs	DC-SIGN^+^	HLA-G1 (57)	n.t.	Pos (57)	n.t.
	DC-10	HLA-G1 (53)	Pos (53)	Pos (53)	n.t.

*The indicated markers have been tested on cells at the maternal–fetal interface and demonstrated to be expressed (Pos) or not (Neg)*.

*The indicated markers have not been tested yet (n.t.)*.

The presence of soluble HLA-G in embryo culture supernatants positively associates with embryo implantation (58–60). The interaction of HLA-G with ILT2 on endometrial stromal cells (47) might contribute to the remodeling of uterine vascularization, and EVT migration and invasion (61, 62). Moreover, the interaction between EVTs and resident dNKs that express both ILT2, although at low levels, and KIR2DL4 (49, 50) guarantees the correct arterial remodeling (Table 1). In contrast to peripheral NK, dNKs are poorly cytotoxic and secrete, in addition to IFN-γ, the pro-angiogenic factors VEGF, placental growth factor (PLGF), angiopoietin 1 and 2, and transforming growth factor (TGF)-β1 (14, 63–66). These molecules promote the uterine vascular changes necessary for maximizing maternal blood flow through the placenta. Moreover, the perivascular localization of dNKs in a microenvironment enriched in EVT-derived soluble HLA-G enables the formation of uterine spiral arteries (67). *In vitro* studies show that the interaction between HLA-G5 and shed HLA-G1, with KIR2DL4 in the early endosome of activated NKs promotes phenotypical and physiological changes leading to cellular senescence, which sustains the secretion of pro-angiogenic mediators (49, 51). Exposure of macrophages (MΦ) isolated from the first trimester decidua to HLA-G-expressing cell lines induces secretion of IL-6, CXCL8, and TNF-α that activate dNK-mediated vascular remodeling (50). Hence, the cross-talk between HLA-G-expressing/secreting EVTs and decidual innate cells coordinate the tissue remodeling necessary for a successful pregnancy.

It cannot be overlooked that EVTs-derived HLA-G also induces tolerogenic immune responses leading to semi-allogeneic fetus acceptance. In addition to dNKs, MΦ, DCs, effector and regulatory T cells, and B cells infiltrate the decidua (52, 68, 69), which are likely to be important determinants in tolerance induction. dMΦ are characterized by low levels of CD86 coupled with the expression of the immunomodulatory molecule indoleamine 2,3-dioxigenase (IDO) (70), and by IL-10 production (50, 71, 72). Gene expression profiling demonstrated that dMΦ from the first trimester of pregnancy express genes functionally related to immunomodulation and tissue remodeling (73). *In vitro* studies showed that exposure of U937 cells to HLA-G5 or HLA-G6 modulates IL-10 and TGF-β secretion (74). Based on these data, and on the fact that dMΦ express ILT2 and ILT4 (50, 56) (Table 1), it was postulated that, in the presence of dNK-derived IFN-γ, dMΦ in contact with HLA-G^+^EVTs and exposed to soluble HLA-G are induced to secrete IL-10 and TGF-β, which limit T-cell responses and promotes tolerance (74).

Plasmacytoid (BDCA-2^+^) and myeloid (BDCA-1^+^ and BDCA-3^+^) DCs have been also identified at the maternal–fetal interface (53, 75, 76). In early human pregnancy, DC-SIGN^+^ dDCs, characterized by low expression of CD86 and DEC-205, were described (77). DC-SIGN^+^ dDCs might be involved in re-programing the local immune response since they are associated with GM-CSF- and IL-10-secreting large granular lymphocytes that inhibit their maturation, and possibly favor tolerogenic responses (78). It has been shown that a population resembling DC-SIGN^+^ dDCs that express ILT4 can be differentiated *in vitro* (57, 76), suggesting that these cells can be also modulated by HLA-G^+^ decidual resident cells (Table 1). Our group identified a peculiar subset of tolerogenic DCs at the maternal–fetal interface in the first trimester of pregnancy. These DCs, termed DC-10, express HLA-G and ILT4 and secrete IL-10, thus are potentially involved in promoting tolerance (53) (Table 1). Future investigation is warranted to define whether dDC-10 and DC-SIGN^+^ dDCs are distinct populations of tolerogenic APCs, or cells at different stages of differentiation.

It is not surprising that Tregs are present in the decidua during pregnancy. An increased frequency of CD4^+^FOXP3^+^ Tregs in the peripheral blood of pregnant women has been shown (79) and the accrual of these cells has been described in human decidua with controversial results (53, 76, 80, 81). Recent evidence indicated that CD4^+^FOXP3^+^ Tregs might be generated *in situ* (57). A population of CD4^+^ T cells expressing HLA-G, termed CD4^+^HLA-G^+^ T cells, representing up to 20% of the decidua-infiltrating CD4^+^ cells, have been recently reported (53, 54) (Table 1).

## Open Issues

### Trophoblast-maternal APCs cross-talk: role of HLA-G-mediated signals

For the acceptance of the semi-allogeneic fetus, a crucial role is played by the trophoblasts themselves. In addition to express/secrete HLA-G, EVTs release immune-modulatory mediators (i.e., IL-10 and TSLP), which are involved in promoting a pro-tolerogenic microenvironment. Our group characterized the tolerogenic DC-10 that are present *in vivo* and are inducible *in vitro* in the presence of IL-10. DC-10 are mature myeloid cells that spontaneously secrete IL-10 in the absence of IL-12, and express HLA-G, ILT2, ILT3, and ILT4. Importantly, DC-10 promote the induction of adaptive T regulatory type 1 (Tr1) cells via the IL-10-induced HLA-G/ILT4 pathway (82). Later, we demonstrated that DC-10 accumulate in human decidua during the first trimester of pregnancy (53). Based on this observation, we postulate that dDC-10 may represent the naturally occurring HLA-G-expressing DCs involved in re-programing the immune response toward tolerance. The recent observation that the frequency of dDC-10 in women with spontaneous abortion is lower compared to that observed in pregnant women sustains this hypothesis (our unpublished data). One of the important questions regarding dDC-10 is whether they are recruited in decidua during pregnancy or are induced *in situ*. Recently, it was demonstrated that the secretion of TSLP by EVTs induces CD11c^+^ dDCs to express co-stimulatory molecules and HLA-DR and to secrete IL-10 and TGF-β (83). TSLP-instructed DCs via TFG-β secretion induce CD4^+^CD25^+^FOXP3^+^ Tregs that inhibit effector T cells, and promote HLA-G expression on EVTs (83). Thus, the decidual microenvironment, enriched in TSLP and IL-10, produced by both EVTs and immune cells, sustains the expression of HLA-G on EVTs. In this scenario, the cross-talk between HLA-G-expressing EVTs and decidual myeloid cells might favor the generation of a set of tolerogenic DCs, including dDC-10 and TSLP-modulated CD11c^+^ dDCs, which co-operate in promoting tolerance via the generation of different subsets of Tregs: Tr1, CD4^+^CD25^+^FOXP3^+^, or CD4^+^HLA-G^+^ cells. As discussed above, EVT-derived HLA-G directs dMΦ toward a tolerogenic path, which contributes to the inhibition of effector T cells and to the induction of Tregs. The hypothesis that decidual tolerogenic APCs drive the differentiation of Tregs is supported by the higher frequency of peripherally induced Tregs (defined as Helios^−^ iTreg) compared to the thymic-derived Tregs in decidua (57). Our group recently demonstrated that co-expression of CD49b and LAG-3 identified Tr1 cells *in vivo* (84); thus, the use of these biomarkers in conjunction with the expression of FOXP3, Helios, and HLA-G will better define Treg cell composition at the maternal–fetal interface and define their relationship and relative contribution in tolerance induction.

Tolerogenic DCs can also contribute to sustain the pro-angiogenic milieu in the decidua. dDC-10 through the HLA-G can interact with dNKs or dMΦ via ILT2 and promote their activation and the release of the angiogenic factors. Moreover, dDC-10 themselves secrete IL-8 and VEGF (our unpublished data), supporting their pro-angiogenic functions. Since dMΦ, dDC-10, and TSLP-modulated CD11c^+^ dDCs are characterized by the ability to secrete IL-10, they can also support the up-regulation of HLA-G on EVTs and on other decidual infiltrating cells (85), hence facilitating the establishment of an appropriate vascular bed at the maternal–fetal interface.

### Trophoblast-decidua cross-talk: role of HLA-G-mediated signals

The pre-decidualization program entails the production of a plethora of transcription factors, cell cycle regulators, cytokines, and the activation of diverse signaling pathways (86). Full decidualization is then achieved upon embryo arrival. In view of the increased requirements for protein secretion during embryo implantation, cytoplasmic and endoplasmic reticulum (ER) stress responses are activated at the maternal–fetal interface. Cytoplasmic stress responses are characterized by the rapid stress-induced synthesis of heat shock proteins (HSPs) that allow cells to restore protein homeostasis and to be protected against molecular damage (87). Stress-induced HSPs are not only essential for regulating the state of intracellular folding, assembly, and translocation of proteins but are also potent modulators of the immune responses. Moreover, HSPs are necessary for placental development. Targeted deletion of HSP90 results in embryonic lethality (88). In primary decidualizing, endometrial stromal cells treated with embryo supernatants, genome wide expression profiling revealed that HSP70 was strongly increased (89).

The range of functions attributed to HSPs has expanded to encompass functions outside the cell (90). Extracellular HSPs may be able to play a role as danger signals (91). In this context, HSPs may interact with pattern recognition receptors, and activate pro-inflammatory signaling and transcription. Specifically, extracellular HSP60 was shown to allow communication between immune cells and other cells in the body (92), and HSP70 can be released from cells after acute stress in different cells, including cultured rat embryo cells (93), and peripheral blood mononuclear cells (94). Notably, HSPs can activate NKs and Tregs (95, 96). Evidence for regulation of HLA-G by HSPs is still scanty. *HLA-G* transcription was found to be induced upon heat shock in tumor cell lines, by heat shock transcription factor 1 (HSF1) binding to a heat shock element (HSE) present in *HLA-G* but not in other *HLA class I* genes (97). Moreover, mice mutant for *Hsf1* have a thin spongiotrophoblast layer and die *in utero* (98). Further investigation is warranted to define if maternal/fetal-derived HSPs might contribute to the regulation of HLA-G expression on dDC-10 and EVTs.

Protein folding in the ER is essential to ensure normal cell function. Disruption of ER homeostasis causes accumulation of misfolded proteins in the ER, a condition referred to as ER stress. ER stress activates the unfolded protein response (UPR) to restore protein homeostasis within the ER. However, if ER stress is persistent and excessive, the ER homeostasis cannot be re-established and the UPR will induce apoptosis. Intriguingly, IL-10 is emerging as a novel modulator of the ER stress (99). Intestinal epithelial cells isolated from IL-10^−/−^ mice exhibit increased expression levels of BiP, a prototypic marker for ER stress, suggestive of an increased ER stress in the absence of IL-10. Further observations revealed that IL-10 attenuates tunicamycin-induced ER stress through suppression of BiP (100). These studies consistently suggest a novel role for IL-10 in modulating ER stress (101). Under ER stress, which occurs during normal development of labyrinthine trophoblasts in the mouse placenta, transcriptional regulation of VEGF is mediated by the three master regulators of the UPR: IRE1a, PERK, and ATF6 (102). The modulation of the UPR pathway by IL-10, produced by dMΦ, dDC-10, and TSLP-modulated CD11c^+^ dDCs, might represent an additional mechanism to regulate vascular uterine remodeling and placentation.

## Perspectives

The existence of mechanisms by which fetal and maternal cells simultaneously attract and modulate each other is intriguing. Upon blastocyst implantation into the uterine wall, trophoblasts differentiate into EVTs that possess the ability to coordinate the cross-talk at the interface via the expression of HLA-G. Accumulating evidence indicate that EVTs play a key role in orchestrating a number of molecular and cellular decidual modifications by (i) regulating cell-migration in the decidua, (ii) supporting the induction of the pro-angiogenic decidual microenvironment necessary for effective vascular remodeling, (iii) inhibiting effector innate and adaptive immune responses, and (iv) promoting a tolerogenic loop in which resident cells are instructed to become tolerogenic. These functions are regulated through the finely tuned specific interactions of HLA-G-expressing EVTs with maternal innate immune cells, adaptive immune cells, and non-immune cells (Figure 1). The interplay among these cells supports the development of an appropriate maternal–fetal niche. Pregnancy hormones are essential to fully support the niche, although their role in regulating HLA-G expression has not been investigated yet (29).

We suggest that the integration and exchange between fetal and maternal blood vessels at the interface is likely to be contributed by multiple mechanisms, including trophoblast interaction with dNKs and resident/recruited APCs, as well as by the IL-10-driven tolerance and regulation of the UPR pathway in decidual and trophoblast cells.

## Conflict of Interest Statement

The authors declare that the research was conducted in the absence of any commercial or financial relationships that could be construed as a potential conflict of interest.
